# Identification and Antibacterial Characterization of Endophytic Fungi from *Artemisia sieberi*

**DOI:** 10.1155/2021/6651020

**Published:** 2021-03-05

**Authors:** Ibtisam Mohammed Ababutain, Sahar Khamees Aldosary, Amal Abdulaziz Aljuraifani, Azzah Ibrahim Alghamdi, Amira Hassan Alabdalall, Eida Marshid Al-Khaldi, Sumayh A. Aldakeel, Noor B. Almandil, Sayed AbdulAzeez, J. Francis Borgio

**Affiliations:** ^1^Department of Biology, College of Science, Imam Abdulrahman Bin Faisal University, P.O. Box 1982, Dammam 31441, Saudi Arabia; ^2^Basic & Applied Scientific Research Center (BASRC), Imam Abdulrahman Bin Faisal University, P.O. Box 1982, Dammam 31441, Saudi Arabia; ^3^Department of Genetic Research, Institute for Research and Medical Consultations (IRMC), Imam Abdulrahman Bin Faisal University, Dammam, Saudi Arabia; ^4^Department of Clinical Pharmacy Research, Institute for Research and Medical Consultations (IRMC), Imam Abdulrahman Bin Faisal University, Dammam, Saudi Arabia; ^5^Department of Epidemic Diseases Research, Institute for Research and Medical Consultations (IRMC), Imam Abdulrahman Bin Faisal University, Dammam, Saudi Arabia

## Abstract

Endophytic fungi serve as a reservoir for important secondary metabolites. The current study focused on the antibacterial properties of endophytic fungi isolated from *Artemisia sieberi*. Initially, six endophytic fungi were isolated and purified from the stem of *A. sieberi*. Endophytic fungi were identified by morphological characteristics, as well as by molecular identification using 18S rRNA gene sequencing method. All the six isolates were subjected to the preliminary screening for their antibacterial activity against nine important pathogenic bacteria using the disk-diffusion method. Crude extracts of the most active isolate were obtained using ethyl acetate. Antibacterial activity of the ethyl acetate extract was evaluated using well diffusion method on the selected isolate. The antibacterial efficiency of the selected isolate was evaluated by determining the Minimum Inhibitory Concentration (MIC). MIC values were in appreciable quantity against both Gram-positive and Gram-negative bacteria ranging from 3.125 to 6.25 *µ*g/mL and 12.5 to 50 *µ*g/mL, respectively. This result indicated that Gram-positive bacteria were more susceptible to the endophytic fungi extract. Moreover, the molecular identification results revealed that all the isolates belong to Ascomycota and represented *Aspergillus* and *Penicillium* genera and three species: *A. oryzae* (three isolates), *A. niger* (one isolate), and *P. chrysogenum* (two isolates). All six endophytic fungi were able to inhibit the growth of at least two of the tested bacteria. Among the isolated strains, isolate AS2, which identified as *P. chrysogenum*, exhibited the highest antibacterial activity against all nine tested bacteria and was higher than or equal to the positive control against most of the tested bacteria. Future studies are required to isolate and identify these bioactive substances, which can be considered as a potential source for the synthesis of new antibacterial drugs to treat infectious diseases.

## 1. Introduction

Endophytic fungi reside in the inner tissues and inside the plant cell of their hosts without causing overt symptoms and damage [1]. Studies suggest that there is a mutualistic interaction between the host and the endophytic fungi, in which the host provides shelter and nutrition, while endophytes act as chemical guards [2, 3]. Research carried out so far regarding the role of endophytes in host plants indicates that they can stimulate plant growth, increase disease resistance, and improve the plant's ability to withstand environmental stress [4, 5]. Endophytic fungi are interesting due to their potential as a source of secondary metabolites and have proven useful for novel drug discovery [6, 7]. There are several studies reporting of antimicrobial activity from plant's endophytic microorganisms [8, 9].

Endophytic fungi are generally considered superior because of their ubiquitous and diverse nature [10]; they produce a large number of secondary metabolites greater than other endophytic microorganisms [11]. Endophytic fungi have drawn interest from natural product chemists in the search for antimicrobial or other active compounds [12]. Medicinal plants are known to harbor endophytic microorganisms, which are found to play an important role in the production of pharmaceutically important compounds [11]. Researchers found that there were large quantities of bactericidal, fungicidal, and cytotoxic metabolites produced by endophytic fungi isolated from medicinal plants [13].

*Artemisia* is a genus of small herbs and shrubs found in temperate regions, belonging to the family Asteraceae, comprised of about 1,000 genera and over 20,000 species. The genus *Artemisia* has about 500 species distributed manly in three continents: Asia, Europe, and North America [14–16]. Moreover, the genus *Artemisia* is raked first for its bioprospection [17].

This genus is one of the most popular plants in traditional medicines and is frequently used for the treatment of several diseases such as aging-related disorders, cancer, diabetes, hepatitis, malaria, obesity, inflammation, and infections by fungi, bacteria, and viruses [18, 19]. Several studies found that the essential oil extracted from *A. sieberi* possesses high antibacterial activities against common human pathogens [20, 21].

The aims of this study were to isolate and identify the endophytic fungi from *Artemisia sieberi* desert plant, which grows in the Eastern province of Saudi Arabia and to investigate the antibacterial activity of these fungi against some important pathogenic bacteria.

## 2. Materials and Methods

### 2.1. Isolation of Endophytic Fungi

Symptomless and mature *Artemisia sieberi* plants were selected for sampling at Eastern province of Saudi Arabia. Twelve stem samples of *A. sieberi* plant were collected and washed thoroughly in running tap water followed by washing with sterile distilled water. The small fragments of the stem were cut with the help of a sterilized razor blade [22] and the surfaces were sterilized with 70% ethanol for 10 min. Then, these sterilized samples were rinsed individually three times in sterile distilled water for 1 min, to remove the excess surface sterilants. The excess moisture was air-dried under sterile conditions. Surface sterilized explants, thus obtained, were evenly spaced in Petri dishes containing Agar medium amended with streptomycin 50 *μ*g/ml-1 to eliminate bacterial growth. Petri plates were sealed with Parafilm and incubated at 28°C until fungal growth started. The cultures were monitored every day to check for the fungal growth indicated by the hyphae emerging from the inoculated segments. The hyphal tips which grew out were subcultured onto Sabauraud's Agar (SDA) plates until pure cultures were obtained.

### 2.2. Preservation and Identification of Endophytic Fungi

Pure fungal cultures were maintained on SDA slants at 4°C and preserved in glycerol at −80°C. Identification of endophytic fungi up to the genera level was carried out using morphological microscopic characteristics (Olympus, USA, at 40X) [23].

### 2.3. 18S rRNA Gene Sequencing

Molecular identification was carried out using *18S rRNA* gene sequencing to identify selected fungal species. Total genomic DNA was extracted from the endophytic fungi samples using the Genomic DNA Purification Kit (Promega, USA) according to the manufacturer. Briefly, cells were filtered through 0.2 *µ*m filters and grinded by freezing with liquid nitrogen. Then cells were incubated with lysis and protein precipitation solutions and eventually the extracted DNA was washed and rehydrated by a rehydration solution with incubation at 65°C for 1 hour. The quality of the isolated DNA was checked using agarose gel electrophoresis and NanoDrop 2000c spectrophotometer (Thermo Scientific, USA). The following primers were used for the amplification of the *18S rRNA*: (*18S rRNA* forward: 5′-GCTTAATTTGACTCAACACGGGA-3′ and 18S rRNA reverse: 5′-AGCTATCAATCTGTCAATCCTGTC-3′). Amplification was carried out in a thermocycler (BioRad, USA) with the following conditions: 95°C for 10 min, followed by 35 cycles of 95°C for 1 min, 67.7°C for 1 min and 15 sec and 72°C for 2 min, and finally an extension step at 72°C for 5 min. The amplified PCR products were purified using a QIAquick PCR Purification Kit (Qiagen, Germany). The purified PCR product was cycle-sequenced using BigDye Terminator Cycle Sequencing Kit (Applied Biosystems, Life Technologies Corporation, USA). The purified cycle sequence products were electrophoresed in Genetic Analyzer 3500 (Applied Biosystems, Life Technologies Corporation, USA) using POP 7. The BLASTn was used through FungiDB to identify the name of the fungi based on the 18S ribosomal RNA gene sequence.

### 2.4. Molecular Phylogenetic Analysis by Maximum Likelihood Method

The evolutionary history was inferred by using the Maximum Likelihood method based on the Tamura-Nei model [24]. The tree with the highest log likelihood (−1516.91) is shown. Initial tree(s) for the heuristic search were obtained automatically by applying Neighbor-Join and BioNJ algorithms to a matrix of pairwise distances estimated using the Maximum Composite Likelihood (MCL) approach and then selecting the topology with superior log likelihood value. The tree is drawn to scale, with branch lengths measured in the number of substitutions per site. The analysis involved 84 nucleotide sequences. Codon positions included were 1st + 2nd + 3rd + Noncoding. All positions containing gaps and missing data were eliminated. There were a total of 610 positions in the final dataset. Evolutionary analyses were conducted using MEGA7 [25].

### 2.5. Preliminary Screening for Antibacterial Activity

Six endophytic fungi isolates (AS1, AS2, AS3, AS4, AS5, and AS6) were screened for their antibacterial activity using the disk-diffusion method [26], against the nine tested bacteria: *Acinetobacter baumannii* ATCCmra747, *Enterobacter aerogenes* ATCC13048, *Escherichia coli* ATCC25922, *Klebsiella oxytoca* ATCC700324, *Klebsiella pneumonia* ATCC100324, *Pseudomonas aeruginosa* ATCC27853, *Staphylococcus aureus* ATCC24213, *Streptococcus agalactiae* ATCC12336, and *Staphylococcus epidermis* ATCC12228, which were obtained from King Fahd Hospital, Al Khobar. Saudi Arabia. Endophytic fungi were cultured on SDA media at 28°C for 7 days. Suspensions of the tested bacteria were prepared from overnight cultures in nutrient broth. The turbidity of the suspension was adjusted to 1.5 × 10^8^ CFU/mL using 0.5 McFarland standards and 100 *μ*l tested bacteria suspension was added to the Nutrient Agar medium (NA) plates. A sterile swab was used to evenly distribute the tested bacteria over the NA medium. The seeded plates were allowed to dry for 15 minutes. Agar plugs (6 mm in diameter) of growing endophytic fungi culture were added to NA plates previously seeded with test bacteria. Disc size 6 mm was loaded with 100 *μ*l of Ampicillin (0.05 mg/mL), which was used as positive control, whereas nutrient broth was used as negative control. Plates were incubated at 37°C for 24 h, and the inhibition zones around the agar plugs were measured to record the antibacterial activity of fungal isolates.

### 2.6. Fermentation and Extraction

Based on the results from preliminary screening of potent antibacterial activity, one endophytic isolate AS2 was selected for further studies. The inoculum was prepared by inoculating 1 cm^2^ mycelia agar plugs age 7 days into two 1000 mL Erlenmeyer flasks, each containing 500 mL of the SD broth medium. The cultures were cultivated at 28°C with speed of 150 rpm. After three weeks of incubation, the fermented broth and fungal biomass were separated out by centrifugation for 15 min. Supernatant was then extracted thrice with equal volume of ethyl acetate (1 : 1, v/v). The upper organic phase was concentrated to dryness under reduced pressure to obtain the crude organic extract. The crude organic extract of the isolate AS2 was kept at 4°C [27].

### 2.7. Well Diffusion Assay

Antibacterial activity of selected endophytic isolate AS2 was carried out using the modified well diffusion method [28]. Three wells were created in each plate previously incubated with the 100 *μ*l of tested bacteria at concentration of 0.5 × 10^8^ CFU/ml using 0.5 McFarland standards and 100 *µ*l of semisolid crude organic extract. The AS2 isolate was dissolved in 5 ml of dimethyl sulfoxide (DMSO) to give a final concentration of 20 mg/ml. Then 100 *µ*l of the late test solution was placed in one of the wells; the other two wells were served as positive and negative controls with 100 *μ*l of Ampicillin (0.05 mg/ml) and 100 *µ*l of DMSO, respectively. The plates were incubated at 37°C for 24 hours after which they were examined for inhibition zones. Experiments were performed in triplicate to ensure reliability.

### 2.8. Determination of Minimum Inhibitory Concentration

The determination of Minimum Inhibitory Concentration **(**MIC) of the selected endophytic isolate, AS2, was carried out using the 96-well microtitre plate's method [29]. Twofold dilution of Ampicillin (0.05 mg/ml) and the endophytic extract was made with Mueller-Hinton Broth (MHB). Fifty microliters of the tested bacteria at concentration of 0.5 × 10^8^ CFU/ml was added to the previous wells. Wells numbers 11 serve as positive control containing MHB media and bacterial inoculum whereas wells numbers 12 serve as negative controls. Two negative controls were used MHB media with endophytic extract and MHB media with Ampicillin. The plates were incubated at 37°C for 24 hours after which they were read using a microtitre plate's reader at a wavelength of 660 nm. Experiments were performed in triplicate to ensure reliability.

## 3. Results

### 3.1. Isolation and Identification of Endophytic Fungi

**Figure 1:**
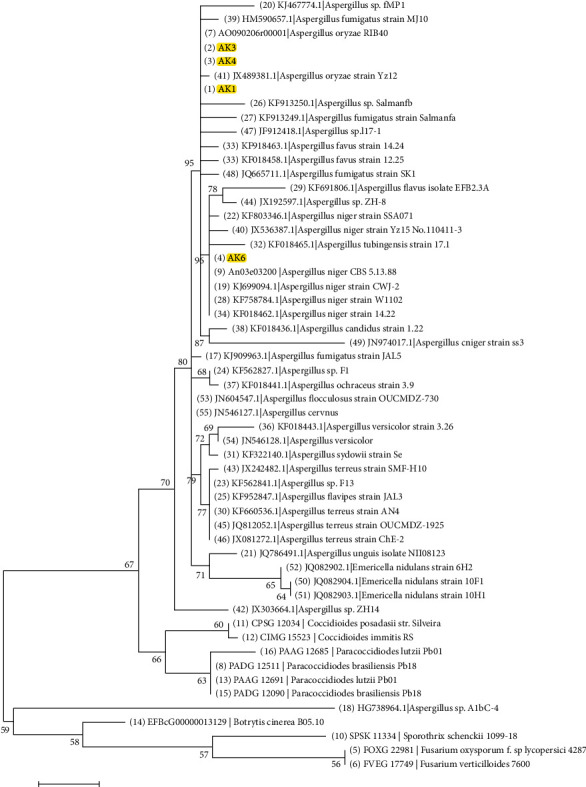
Phylogenetic trees constructed from partial *18S rRNA* gene sequence of strains AK1, AK3, AK4, and AK6 representing AS1, AS3, AS4, and AS6, respectively. The locations of the strains AK1, AK3, AK4 (named *Aspergillus oryzae*), and AK6 (named *Aspergillus niger*) are indicated by the yellow highlight.

**Figure 2:**
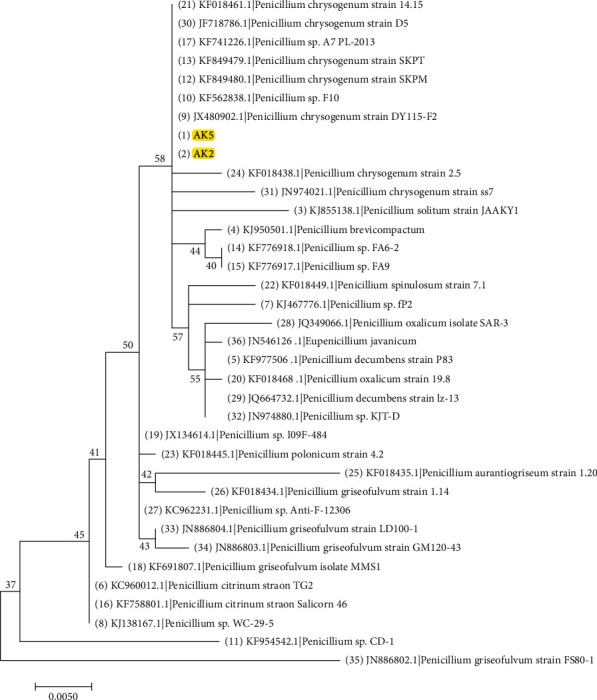
Phylogenetic trees constructed from partial *18S rRNA* gene sequence of strains AK2 and AK5 representing AS2 and AS5, respectively. The locations of the strains AK2 and AK5 (named *Penicillium chrysogenum*) are indicated by the yellow highlight.

The stem of *A. sieberi* was colonized by endophytic fungi, from which six endophytic fungi were isolated. Based on the investigated structural morphology, all of the isolates belonged to two genera: *Aspergillus* and *Penicillium* of Ascomycota phylum*. 18S rRNA* gene sequencing reveled that endophytic fungi represented three species *A. oryzae*, *A. niger*, and *P. chrysogenum*, representative 18S rRNA gene sequences were submitted to GenBank and the accession numbers were received (Table 1; Figures 1 and 2).

### 3.2. Preliminary Screening for Antibacterial Activity

The antibacterial screening results showed that all six endophytic fungi were able to inhibit the growth of at least two of the tested bacteria. Isolate number AS2 was able to inhibit the growth of all nine tested bacteria. The tested bacterium *S. aureus* ATCC24213 was the most sensitive bacterium toward all endophytic fungi isolates (Table 2).

### 3.3. Well Diffusion Assay

The results of well diffusion assay showed that the crude extract of the endophytic isolate, AS2, highly inhibited the growth of all tested bacteria. Moreover, the inhibitory activity of the isolate AS2 was higher than the positive control against some of the tested bacteria such as *E. aerogenes* ATCC13048, *K. oxytoca* ATCC700324, and *S. epidermis* ATCC12228 (Table 3).

### 3.4. Determination of Minimum Inhibitory Concentration

The results of MIC showed that the extract of the endophytic isolate, AS2, highly inhibited the growth of all tested bacteria. Moreover, the MIC values of the isolate AS2 extract were equal to the positive control against 5 out of 9 tested bacteria. It is worth noting that the inhibitory capacity of the extract of endophytic isolate AS2 was higher than positive control against 3 out of 9 tested bacteria (Table 4).

## 4. Discussion

Endophytes are known as valuable sources of diverse range of important natural compounds [30–32]. The metabolic compatibility for natural products secretion has been greatly influenced by natural selection [32]. The current study addressed that *A. sieberi* stem is rich in endophytic fungi in which six endophytic fungi were isolated from twelve samples. The results of the current study are consistent with a number of previous studies that isolated other endophytic fungi from different tissues of *Artemisia* species. For instance, 4 endophytic fungi were isolated from *A. annua* leaves [33], and 42 endophytic fungi were isolated from *A. sieberi* stem and root [34]. Moreover, 108 endophytic fungi were isolated from twenty samples of three *Artemisia* species (*A. capillaris*, *A. indica*, and *A. lactiflora*) [35]. These studies indicate that *Artemisia* species are rich in endophytic fungi.

The present study indicated that the fungal diversity was low because all the six isolates belonged to one phylum Ascomycota. There are several factors that influence the diversity of endophytes such as plant age, plant parts, seasonal collection, soil type, geographics, and other environmental conditions [36, 37]. Also, these endophytic isolates represented only two genera, *Aspergillus* and *Penicillium,* which is consistent with other studies, which found that the endophytic fungi are mainly from Ascomycota phylum [34, 38]. The current study showed that, despite the low diversity of isolated fungi, the isolated endophytic fungus *A. niger* was not previously isolated from the genus *Artemisia* or its species.

The current study showed that endophytic fungi isolated from *A. sieberi* stem have the potential to inhibit the growth of at least two of the pathogenic tested bacteria which may be related to the presence of natural bioactive compounds that possessed growth inhibitory activity. This finding is consistent with other studies, which reported that endophytic fungi could be considered as a good source for secondary metabolites that have bioactive compounds [4, 38].

Recently, endophytic fungi have been isolated from some parts of medicinal plants that possessed several active biological compounds such as alkaloids, diphenyl ether, monocarboxylic acid, hydroxycinnamic acid, phenalenones, sterols, terpenoids, and xanthones [39, 40]. Mawabo et al. [41] found that *A. niger* isolated from *Acanthus montanus* stems was able to produce two chemical compounds, namely, Trypacidin A and Methylsulochrin, both of which possess the ability to inhibit tested bacteria with different degrees. Methylsulochrin had antibacterial activity against *Enterococcus faecalis* ATCC51299, *Enterobacter cloacae* BM67, and *Mycobacterium smegmatis* ATCC700084, whereas Trypacidin A inhibited only *Mycobacterium smegmatis* ATCC700084. Noor et al. [42] reported that isocoumarins derivatives which have a wide range of bioactivities are mainly produced by endophytic fungi which belong to *Aspergillus* and *Penicillium* genera. Accordingly, the antibacterial activity of the endophytic fungi isolated in the present study might be associated with the presence of one or more of these bioactive compounds in their raw extracts.

This study showed that the isolate AS2, which was identified as *P. chrysogenum*, exhibited the highest antibacterial activity against all nine tested bacteria that could be a new potential source for a broad spectrum of antibacterial agents. Second highest antibacterial activities were exhibited by AS5 and AS6 isolates, which were identified as *P. chrysogenum* and *A. niger*, respectively, and inhibited three out of the nine tested bacteria. Lastly, AS1, AS3, and AS4 isolates, which were identified as *A. oryzae,* inhibited only two out of the nine tested bacteria. *P. chrysogenum*, *A. niger*, and *A. oryzae* have been shown previously to inhibit the growth of pathogenic bacteria. Gashgari et al. [34] found that *P. chrysogenum* endophytic fungus isolated from some medicinal plants showed the highest inhibitory activity against pathogenic bacteria. *A. niger* isolated from *Hancornia* species displayed excellent inhibition activity against *Proteus mirabilis* and *S. aureus* [12]. Also, Thorati and Mishra [43] found that endophytic fungus *A. niger* isolated from *Rhizophora apiculata* exhibited good inhibition activity against pathogenic bacteria and they related that to the presence of bioactive compounds such as proteins, terpenoids, and saponins. Rani et al. [44] found that *A. oryzae* and *A. niger* isolated from *Calotropis procera* were able to inhibit the growth of both Gram-positive and Gram-negative pathogenic bacteria. Recently, Aruna et al. [45] found that the endophytic *A. oryzae* that was isolated from *Wattakaka volubilis* possessed antibacterial activity toward *Micrococcus luteus* and *E. coli* but was not able to inhibit the growth of *Staphylococcus aureus* and *Klebsiella pneumonia*. The difference in results may be due to plant type, geographical conditions, endophytic extract concentration, pathogenic bacteria strain, and endophytic fungal strain. To measure the antibacterial efficiency of the endophytic extract from the isolate AS2, the MIC was determined. The result showed appreciable MIC values against both Gram-positive and Gram-negative bacteria ranging from 3.125 to 6.25 *µ*g/mL and 12.5 to 50 *µ*g/mL, respectively. These results indicated that Gram-positive bacteria were more susceptible than Gram-negative bacteria to the endophytic extract; this may be attributed to the nature of their cell wall structure [46].

## 5. Conclusion

The present study indicated that *A. sieberi* is rich in endophytic fungi where six endophytic fungi were isolated from twelve stem samples and all the isolates belonged to phylum Ascomycota and represented two genera *Aspergillus* and *Penicillium*. In this study, endophyte *A. niger* was isolated for the first time from *A. sieberi*. Endophytic fungi *P. chrysogenum* inhibited the growth of all tested pathogenic bacteria while the other five isolates were found to inhibit a maximum of two to three of the tested bacteria. It was interesting that these antibacterial fungi could inhibit both Gram-positive and Gram-negative pathogenic bacteria, revealing the possible existence of some broad-spectrum antibacterial properties. It was noteworthy that *P. chrysogenum* isolate not only exhibited broad-spectrum activity but also was competent enough to compare with the positive control. The diameters of their inhibition zones and the MIC values against specific tested bacteria were close to or even higher than those of the positive control suggesting their potential use as new antibacterial agents. Future studies are required to isolate and identify the substance responsible for the inhibition of the pathogenic bacteria to utilize it in manufacturing drugs that could treat infections caused by antibiotic-resistant bacteria.

## Figures and Tables

**Table 1:** Identification of endophytic fungi isolates from *Artemisia sieberi*.

Isolates ID	Morphological identification	Molecular identification using 18S *rRNA* gene sequencing	GenBank accession numbers
AS1/AK118SRNA	White mycelium later became dark brownish on surface side and pale brownish in the reverse	*Aspergillus oryzae*	MH842201
AS2/AK218SRNA	White mycelium later became bluish to dark green on surface side, beige in the reverse and yellowish pigment	*Penicillium chrysogenum*	MH842202
AS3/AK318SRNA	Whitish mycelium later became green brownish on surface side and yellowish in the reverse	*Aspergillus oryzae*	MN528767
AS4/AK418SRNA	Whitish mycelium later became dark brownish on surface side and pale brownish in the reverse	*Aspergillus oryzae*	MN528766
AS5/AK518SRNA	White mycelium later became grayish to dark green on surface side, beige in the reverse and yellowish pigment	*Penicillium chrysogenum*	MN532489
AS6/AK618SRNA	White mycelium later became black on surface side and pale yellowish in the reverse	*Aspergillus niger*	MH842204

**Table 2:** Preliminary screening of the antibacterial activity for the endophytic fungi isolates.

Test bacteria	Inhibition zone (mm) using disk-diffusion method						Ampicillin^1^
	AS1	AS2	AS3	AS4	AS5	AS6	
*A. baumannii* ATCCmra747	0	4.3 ± 0.8	0	0	0	0	5 ± 0.5
*E. aerogenes* ATCC13048	0	3.8 ± 0.3	0	0	0	0	4 ± 0.5
*E. coli* ATCC25922	0	5.8 ± 0.3	3.3 ± 0.6	0	3.2 ± 0.3	3.2 ± 0.3	8.8 ± 0.3
*K. oxytoca* ATCC700324	0	4.0 ± 0.8	0	0	0	0	4 ± 0.5
*K. pneumonia* ATCC100324	0	3.2 ± 0.3	0	0	0	0	4 ± 0.5
*P. aeruginosa* ATCC27853	3.0 ± 0.5	4.0 ± 0.5	0	0	3.3 ± 0.6	3.8 ± 0.8	5.2 ± 0.3
S*. aureus* ATCC24213	4.2 ± 0.3	8.0 ± 0.5	4.0 ± 0.5	3.2 ± 0.3	4.0 ± 0.5	3.2 ± 0.3	10.2 ± 0.3
*S. agalactiae* ATCC12336	0	6.0 ± 0.5	0	0	0	0	9.3 ± 0.6
*S. epidermis* ATCC12228	0	6.2 ± 0.3	0	0	0	0	6 ± 0.5

^1^Positive control. DMSO, negative control = 0.0.

**Table 3:** Antibacterial activity of the endophytic extract from AS2 isolate using well diffusion method.

Test	Inhibition zone (mm) by well diffusion method								
	*Test bacteria*								
	*A. baumannii* ATCCmra747	*E. aerogenes* ATCC13048	*E. coli* ATCC25922	*K. oxytoca* ATCC700324	*K. pneumonia* ATCC100324	*P. aeruginosa* ATCC27853	S*. aureus* ATCC24213	*S. agalactiae* ATCC12336	*S. epidermis* ATCC12228
Isolate AS2	5.0 ± 0.5	6.0 ± 0.5	7.3 ± 0.6	6.0 ± 0.5	3.8 ± 0.3	5.0 ± 0.5	9.2 ± 0.3	9.0 ± 0.5	9.0 ± 0.5

Positive control and negative control: see Table 2.

**Table 4:** Determination of the Minimum Inhibitory Concentration (MIC) *μ*g/ml of the endophytic extract AS2 isolate and Ampicillin against the tested bacteria.

Test	*A. baumannii* ATCCmra747	*E. aerogenes* ATCC13048	*E. coli* ATCC25922	*K. oxytoca* ATCC700324	*K. pneumonia* ATCC100324	*P. aeruginosa* ATCC27853	*S. aureus* ATCC24213	*S. agalactiae* ATCC12336	*S. epidermis* ATCC12228
Endophytic extract	50	25	12.5	25	50	25	3.125	6.25	3.125
Ampicillin	50	50	6.25	50	50	25	3.125	6.25	12.5
Negative control^1^	0	0	0	0	0	0	0	0	0
Negative control^2^	0	0	0	0	0	0	0	0	0

^1^MHB media with endophytic extract, ^2^MHB media with Ampicillin.

## Data Availability

The data presented in this research are available upon request from the institutional archives of the corresponding author.
